# Insight in the Unmet Needs Encountered During the Management of Chronic Inflammatory Demyelinating Polyradiculoneuropathy

**DOI:** 10.1111/jns.70067

**Published:** 2025-10-06

**Authors:** Jeffrey A. Allen, Helmar C. Lehmann, Eduardo Nobile‐Orazio, Luis Querol, Yusuf A. Rajabally

**Affiliations:** ^1^ Department of Neurology University of Minnesota Minneapolis Minnesota USA; ^2^ Department of Neurology Klinikum Leverkusen Leverkusen Germany; ^3^ Medical Faculty University Hospital of Cologne Köln Germany; ^4^ Neuromuscular and Neuroimmunology Unit IRCCS Humanitas Research Hospital Milan Italy; ^5^ Department of Medical Biotechnology and Translational Medicine Milan University Milan Italy; ^6^ Department of Neurology, Neuromuscular Diseases Unit, Hospital de La Santa Creu I Sant Pau (IR SANT PAU) Universitat Autònoma de Barcelona Barcelona Spain; ^7^ Centro de Investigación Biomédica en Red en Enfermedades Raras (CIBERER) Madrid Spain; ^8^ Aston Medical School, School of Health and Life Sciences Birmingham UK

**Keywords:** chronic inflammatory demyelinating polyradiculoneuropathy, diagnosis, responders management, response assessment, therapeutic response

## Abstract

Chronic inflammatory demyelinating polyradiculoneuropathy (CIDP) is a rare acquired immune‐mediated disorder affecting peripheral nerves, manifesting most commonly as symmetric, proximal, and distal weakness with sensory loss. Although the 2021 European Academy of Neurology/Peripheral Nerve Society guidelines provide evidence‐based and consensus‐driven approaches to the diagnosis and treatment of CIDP, challenges to optimal patient care persist. This report aims to highlight the unmet needs in CIDP management. A structured analysis of existing evidence was conducted to map gaps in CIDP care pathways, emphasizing diagnostic criteria, assessment of the therapeutic response, and disease management. Recognized key gaps and unmet needs in CIDP include (1) the absence of specific biomarkers for CIDP, (2) weighing the relative value of various CIDP metrics and interpreting what those metrics say about disease activity and treatment response, and (3) understanding the optimal timing and approach to assess treatment efficacy (or failure). There exists variability in how diagnostic and treatment guidelines are utilized, as well as how (and if) outcome metrics are utilized to guide informed treatment decisions. At least part of the confusion stems from the absence of terms commonly used during the CIDP treatment journey, including “response,” “refractory,” “remission,” and “relapse.” To address these ambiguities, a consensus‐driven effort is needed to establish standardized definitions for key treatment milestones in CIDP. Harmonizing terminology will not only facilitate more accurate clinical assessments but also promote more robust and comparable research outcomes, ultimately improving the care of individuals with CIDP. This report underscores the critical unmet needs in CIDP diagnosis and management. By identifying barriers and facilitators within the current CIDP landscape, we hope to optimize clinical decision‐making and focus research efforts.

**Abbreviations**
Caspr1: contactin‐associated protein 1
CIDP: chronic inflammatory demyelinating polyradiculoneuropathy
CNTN1: contactin‐1
CT‐RC: CIDP treatment‐response category
EAN/PNS: European Academy of Neurology and Peripheral Nerve Society
INCAT: inflammatory neuropathy cause and treatment
I‐RODS: Rasch‐built Overall Disability Scale
IVIg: Intravenous immunoglobulin
MCID: minimal clinically important differences
NCS: nerve conduction study
NF155: neurofascin‐155
SCIg: subcutaneous immunoglobulin
TUG: time up‐and‐go

## 1 Introduction

Chronic inflammatory demyelinating polyradiculoneuropathy (CIDP) is a rare acquired immune‐mediated disorder affecting peripheral nerves and nerve roots, with an estimated prevalence of 2.81 cases per 100 000 individuals [1]. The disease typically manifests as symmetric, proximal and distal weakness with sensory loss in the upper and lower limbs and decreased or absent deep tendon reflexes on examination. The manifestations of CIDP may present insidiously or acutely and are progressive or relapsing over more than 8 weeks. Variants of CIDP may differ in clinical features and include multifocal or focal CIDP, distal CIDP, motor CIDP, and sensory CIDP [2, 3, 4]. Like “typical” CIDP, all variants are defined by the presence of numbness and/or weakness but may differ on the location or type of modality that is affected.

The European Academy of Neurology and Peripheral Nerve Society (EAN/PNS) published updated guidelines in 2021 to enhance diagnostic precision and facilitate earlier identification of CIDP and its variants [4]. The guidelines also describe evidence‐based treatment medications, including recommendations for the use of intravenous immunoglobulins (IVIg), subcutaneous immunoglobulins (SCIg), corticosteroids, and plasma exchange, as induction treatment or maintenance treatment [4]. However, despite these advancements, challenges in diagnosis and treatment persist.

This report aims to highlight the unmet needs in CIDP. To achieve this goal, a group of five CIDP experts considered existing literature and guidelines and offered their viewpoint on the critical gaps that are encountered during the diagnosis and management of CIDP patients.

## 2 Diagnosis

Reaching an early and accurate diagnosis of CIDP can be challenging. CIDP is without a diagnostic biomarker, and consequently, the diagnosis relies upon identifying the characteristic clinical features of the disease and integrating them with key electrophysiologic and laboratory data. Despite recent advances in diagnostic criteria, such as the 2021 EAN/PNS guidelines, misdiagnosis is common due to a complex interplay of clinical evaluations and electrophysiological tests [5, 6]. When used as intended, the guidelines have been demonstrated to have a favorable sensitivity and specificity profile but are criticized for their complexity and are underutilized in many routine clinical care settings. This observation underscores the need to find a more accessible approach to rapid and accurate integration of clinical findings and diagnostic assessments [7].

One significant gap is the absence of specific biomarkers for CIDP. About 5%–10% of patients harbor antibodies to nodal and paranodal cell‐adhesion molecules (contactin‐1 [CNTN1], neurofascin‐155 [NF155], contactin‐associated protein 1 [Caspr1], and neurofascin isoforms NF140/186), but in the majority of patients, no specific antigenic target is known [4, 8]. Although in some ways nodal/paranodal positive patients resemble typical CIDP, many have characteristic clinical features (such as tremor and ataxia, or early aggressive onset) and treatment responses (poorly immunoglobulin responsive) that set them apart from antibody negative CIDP patients. The unique clinical, immunobiological, and treatment features of nodal/paranodal positive patients have led to a separate diagnostic classification distinct from CIDP, referred to as autoimmune nodopathies according to the EAN/PNS guidelines. This shift in classification emphasizes the need for refined diagnostic criteria that account for varying antibody profiles. This is also the case for paraproteinemic neuropathy with anti‐MAG antibodies, where the presence of high anti‐MAG antibody titre (> 7000 Bühlmann units) may indicate an alternative diagnosis with different treatment responses [4, 7].

Another significant gap is the reliance on nerve conduction studies (NCS) as the gold standard for diagnosis. Although these studies play a key role in characterizing nerve pathophysiology as demyelinating, they are not specific for CIDP as not all demyelinating neuropathies are CIDP. NCS may also fail to capture demyelinating changes in a nerve if pathology is inaccessible to an NCS or if nerve injury has advanced beyond pure demyelination [4]. NCS guidelines for CIDP are also criticized for their complexity and difficulty incorporating into day‐to‐day clinical care [9].

In summary, the unmet needs in CIDP diagnosis include the need for specific diagnostic biomarkers to differentiate it from other neuropathic disorders and refined classification systems based on antibody testing. Addressing these gaps is crucial for advancing patient care and optimizing treatment outcomes in CIDP. To address these challenges, several solutions may provide a path forward. Continued investment in biomarker research is essential, with collaborative multicenter studies aimed at identifying novel, disease‐specific biomarkers that can distinguish CIDP from other neuropathies with greater precision. Simplifying and streamlining diagnostic criteria for use in routine clinical practice could enhance their uptake, possibly through digital tools or standardized app‐based checklists that guide clinicians step by step. Additionally, expanding education and training for healthcare providers on the nuances of CIDP diagnosis and the correct interpretation of electrophysiologic and serologic findings may reduce misdiagnosis rates. Finally, fostering partnerships between academic centers, patient advocacy groups, and industry can accelerate access to advanced diagnostic technologies and support the development of evidence‐based guidelines that evolve with scientific advances.

## 3 Assessing the Therapeutic Response

The EAN/PNS guideline provides a framework for diagnosis and treatment and also advises on metrics that can be used in daily clinical practice to assess the treatment response [4]. A comprehensive approach combining objective and subjective measures is essential for accurately evaluating clinical change and, to that end, the guideline highlights key measures of disability and impairment that can be informative when used in daily practice [10].

The absence of a consensus treatment sequence complicates management. First, there is a lack of standardized guidance for treatment initiation. Current literature provides no comparative data on the superiority of corticosteroids, IVIg, or plasma exchange as first‐line therapy, leading to variability in clinical practice [11, 12]. Also, no validated algorithm exists to guide escalation to second‐line agents like rituximab or cyclophosphamide in refractory cases, the evidence supporting these treatments being very limited, primarily derived from case series, which suggest caution. Treatment choices are often influenced by comorbid medical conditions, the response to previous treatments, ease of administration, cost considerations, and patient preferences [4].

The guideline offers guidance on minimal clinically important differences (MCIDs) of each outcome that can serve as a benchmark to understand if a change in any outcome is a meaningful change. Although no MCID perfectly captures the outcome in all CIDP patients, the recommended metrics and MCIDs within the guideline are derived from evidence‐based methodology and have been successfully used as outcome measures in clinical trials. Furthermore, most of the disability and impairment metrics are free to use, available in the public domain, and are not time‐consuming. Disability can be quantified in CIDP patients by using the Inflammatory Neuropathy Cause and Treatment (INCAT) scale and the Rasch‐built Overall Disability Scale (I‐RODS), while strength impairment may be assessed with the MRC sum scale and handheld grip strength. A 1‐point change on the INCAT scale and a 4‐point change on the I‐RODS scale are considered to be the MCID for disability metrics [13]. Individualized MCIDs have also been used, with the advantage of taking into consideration the pre‐treatment disability level [14]. For strength impairment, a 2–4‐point change on an MRC 60‐point sum score is considered clinically meaningful, while grip strength changes of 8 kPa or more are meaningful when assessed with the Martin Vigorimeter. The guideline highlights the importance of having complementary metrics of different domains. If improvement after treatment is appreciated on at least one disability and one impairment scale, then one may be more confident in the therapeutic response [4, 13, 15]. Although outcomes such as these are traditionally reserved for research purposes, we have found them invaluable during the routine evaluation of patients in the clinic. The value of data collected easily justifies the minimal time needed to collect the assessments.

Despite their unequivocal value in clinical trials and clinical practice, there is room for outcomes improvement. First, although the MCIDs provided in the guideline can be used as a framework to assess responses to treatment, the sensitivity and specificity of individual patients may be suboptimal. Changes less than the MCID may be clinically meaningful in some patients, while changes that exceed the MCID may be less informative if secondary factors influence the score. Second, there is no standardization across clinical practice regarding which assessment tools should be uniformly employed [16]. Third, the clinical heterogeneity of CIDP makes it unlikely that any outcome will capture meaningful changes in all patients across the CIDP spectrum. For example, while the MRC sum score may be a good way to capture change in a patient with “typical” CIDP or “motor” CIDP, in distal and sensory variants, it may be less helpful [6]. For this reason, the guideline highlights the importance of multiple domain assessment when assessing the treatment response. Fourth, therapeutic goals are often ambiguous, which can lead to a failure to adjust treatment if a poorly defined objective is not reached. The failure of a healthcare provider to escalate treatment when a patient is not adequately responding, also known as therapeutic inertia, may in part be attributed to the way “response” is defined or measured [17]. Thus, a comprehensive composite measure that addresses all aspects of disability and impairment experienced by CIDP patients is desirable but, at present, unknown [18].

In addition to traditional metrics of disability and impairment used in clinical trials, other patient‐reported outcomes (PROMs) may be helpful to quantify treatment efficacy in day‐to‐day practice. Scales that assess pain, fatigue, and overall well‐being may be constructive to assess in selected patients [19, 20]. The I‐RODS scale, for example, provides a broad assessment of functionality in patients with CIDP, but may also be responsive to other comorbidities and lifestyle factors. Gait assessments, including time‐up‐and‐go (TUG) or 10‐m walk, can provide objective data on gait impairment in CIDP. Although both require validation in CIDP, changes in TUG as small as 1 s may be meaningful [21].

Disease activity biomarkers responsive to improvement or relapse are needed to improve our ability to detect change in patients with CIDP. Neurofilament light chain, although nonspecific, is one candidate that appears to reflect disease activity in untreated patients but is insensitive to real‐time clinical changes for patients on chronic therapy. Nodal/paranodal autoantibodies, such as neurofascin‐155 and contactin‐1, are instrumental diagnostic tools and may reflect disease activity posttreatment but represent only a small proportion of the acquired immune‐mediated polyneuropathy population. These patients are now excluded from the diagnosis of CIDP. Reliable biomarkers for disease monitoring remain elusive for the vast majority of patients with CIDP [22].

In summary, disability and impairment outcomes are key tools that can be used in day‐to‐day practice in order to more confidently assess the treatment response. While the EAN/PNS guideline provides recommendations for how these metrics might be applied, further research is needed to better understand how specific outcomes should be interpreted in the context of each unique patient's circumstances. MCIDs, while informative on a group level, may not be specific or sensitive on an individual patient level. To address these challenges, large‐scale, multicenter studies that collect standardized outcomes across the full spectrum of patient experiences and disease subtypes are needed. INCBase, a worldwide CIDP registry, is currently enrolling and may be one resource that can address this issue. Additionally, when assessing treatment response or attempting to assess disease activity, it is essential to incorporate both patient‐reported outcomes and objective clinical measures, as this may help tailor MCID thresholds to individual patients. Periodic re‐evaluation and validation of these metrics in real‐world clinical settings are needed to ensure that MCIDs remain relevant and truly reflective of meaningful changes in patients' lives. The generation of a composite score that incorporates both the patient experience and MCIDs from several domains may provide an even more holistic assessment of therapeutic response by integrating formal assessment of disability and impairment with the patient voice. Investing in collaborative research focused on identifying reliable, disease‐activity biomarkers has the opportunity to provide the largest advancement for monitoring disease activity. Although a biomarker in isolation may have limited value, if incorporated into a composite assessment, a biomarker may be especially powerful to support treatment response or disease activity.

## 4 Considering Dose and Timing When Assessing the Therapeutic Response

Prompt assessment of treatment efficacy is crucial, as an inadequate response often necessitates a reevaluation of both the CIDP diagnosis and treatment approach [6]. However, the optimal timing and strategy to evaluate treatment efficacy are unknown and may well be variable from patient to patient and from therapy to therapy [4, 23]. This section addresses the complexities of determining assessment timing, emphasizing the need to balance timely intervention with allowing sufficient time for treatment effects to manifest.

CIDP treatment response rate varies between 54% and 92% for IVIg, and is generally about 60% for corticosteroid [24, 25, 26]. The onset of benefit to IVIg is typically within 3 months and almost always within 6 months. The time to reach a maximum improvement is often much longer. Although about 20% of patients reach their maximum improvement at 3 months, 60% may take up to 12 months, and some may take up to 36 months, suggesting transient refractoriness rather than absolute non‐response [17]. The strategy to best identify first benefit and maximum benefit is unknown. Even more challenging is knowing when maximum benefit is reached when residual symptoms and signs persist. Conversely, it is equally important to know when benefit has not occurred and to understand when treatment should be considered ineffective and aborted.

Nonresponse is often defined as lack of improvement in outcomes after first‐line therapies, such as IVIg, corticosteroids, or plasma exchange [27]. This definition, however, is not universally applied. In some settings, improvement with residual neurologic deficits is considered a non‐response, while in other settings, poor treatment tolerability is considered nonresponse. The implications of how this definition is interpreted can be substantial, as a complete lack of clinical change after treatment may prompt diagnostic re‐exploration or consideration of an escalated immunotherapy approach, while a partial or incomplete response may still support the diagnosis and ongoing treatment.

In the event that no beneficial response is appreciated, current data do not establish a definitive timeline for declaring a treatment failure. While the timing of first response may vary depending on the specific therapeutic initiated, in our view if no response is appreciated by 3 months and always by 6 months, then the therapy is unlikely to have a meaningful beneficial effect in that patient [27].

In addition to ensuring that adequate duration has been trialed before considering a treatment to be a failure, it is also important to consider the dose of the selected therapeutic. Practitioners may frequently label patients as refractory based on suboptimal steroid or immunoglobulin regimens despite evidence that higher‐dose therapy may yield better responses [15, 27]. The 2021 EAN/PNS guidelines emphasize verifying adequate dosing and duration before classifying nonresponse [4]. For IVIg, the guideline recommends a loading dose of 2 g/kg over 2–5 days followed by a maintenance dose of 1 g/kg every 3 weeks. Corticosteroids may be administered as daily oral or pulse doses. Oral prednisone typically begins at 0.75–1.5 mg/kg/day and is gradually tapered over several months based on clinical response. The oral dexamethasone regimen is 40 mg daily for 4 consecutive days per month for up to 6 months, and pulse methylprednisolone is 500–1000 mg intravenously daily for 3–5 consecutive days repeated weekly or monthly. The guidelines advise that plasma exchange be administered as 5 exchanges (usually 40–50 mL plasma/kg per session) over 2 weeks as an initial therapeutic regimen, followed by individual adaptation depending on the durability of the treatment effect.

In cases of nonresponse, second‐line treatments like cyclophosphamide or rituximab may be considered; however, clearer guidelines on when to escalate treatment are needed. Without fully understanding how to measure disease activity, it is challenging to know if a patient that fails to respond to treatment does so because the residuals are irreversible from chronic axon loss but are not immunologically active, or if the residuals reflect potentially salvageable deficits if the immunotherapy plan were escalated. Balancing the risks and uncertain benefits in these patients is challenging, especially in the absence of clinical trial data that clearly supports the use of off‐label immunotherapies [4, 28, 29]. Especially considering the risks and unknown benefits of off‐label therapeutics, in the event that a patient is considered to be refractory to a first‐line therapy, a systematic diagnostic reassessment should be undertaken to ensure that the presumed diagnosis of CIDP is accurate. Just as objective improvement following treatment with immunotherapy supports a diagnosis of CIDP, the absence of benefit makes a CIDP diagnosis substantially less likely [30].

The timing of therapeutic response assessment in CIDP hence requires a balanced approach that considers both prompt evaluation and the potential for delayed responses [31]. To address the issues of dose and timing, more sophisticated tools are needed to detect the earliest possible benefit, as this would support ongoing utilization of that therapeutic until a maximum benefit is reached. Such tools may include identification of tissue status or effector mechanism biomarkers, or may include composite clinical outcomes that can more confidently detect a treatment response (or nonresponse). In the case of off‐label therapeutic usage, additional clinical trials are needed to better understand the effectiveness of those therapies in CIDP or subsets of CIDP variants. Finally, while the CIDP diagnostic guideline provides a valuable resource to guide treatment initiation and escalation, this document will require periodic revision to meet the ends of an expanding therapeutic landscape.

## 5 Challenges Encountered During Maintenance Therapy

While achieving an initial treatment response is essential, it has been well recognized that not all patients with CIDP need long‐term immunotherapy. The best approach to optimize immunotherapy, or to understand when and if treatment can be withdrawn, has not been clearly defined. Increasing or decreasing immunotherapy to find the most optimal result relies on clinical judgment.

Decisions regarding treatment de‐escalation or discontinuation are complex and lack standardized guidelines. One study found that approximately 40% of subjects could discontinue treatment without deterioration [17]. This observation reflects the experience of most CIDP randomized placebo‐controlled trials in which approximately 40% of patients do not relapse when randomized to placebo. The challenge becomes identifying these patients. The optimal duration of maintenance therapy for any given patient is uncertain; some patients may require ongoing treatment while others might safely discontinue it. While withdrawing therapy is desirable in those with inactive disease, the risk of relapse and accumulation of additional neurologic impairments cannot be dismissed. Furthermore, although guidelines advocate periodic immunotherapy taper attempts and the usage of the lowest effective therapeutic dose, the best strategy to achieve immunotherapy withdrawal or optimization is unknown.

A separate but related issue is how best to escalate immunotherapy in partially responding patients. In the absence of randomized controlled trials, the “next best” therapy in a refractory or partially responsive patient must consider the patients' tried and failed therapies, comorbid conditions, and risk assessment. CIDP is felt to be immunobiologically heterogenous but has uncertain immunobiological underpinnings. The mechanism of action of different interventions also should be considered when escalating treatment options. If, for example, an antibody clearing strategy was ineffective, an agent that interacts with cellular immunity may be a logistical next step. Such considerations will become more and more important as therapeutics gravitate toward targeted mechanisms of action.

The most pressing ongoing gaps in long‐term disease management include lack of precise definitions of remission, standardized protocols for treatment de‐escalation and discontinuation, and strategies capable of inducing durable treatment response or cure. To address these limitations, widespread adoption of existing standardized treatment guidelines during routine clinical care is a useful first step. As new data comes forth from randomized controlled trials, periodic guideline updates will be needed to keep pace with the evolving evidence‐based treatment landscape. Innovative studies are needed to better understand how best to optimize or withdraw current therapies, including immunoglobulin, corticosteroids, and FcRn antagonist. Regarding definitions of remission, relapse, and residual deficits, a consensus‐based approach may be best suited to establish agreed‐upon definitions that can be used in clinical trials and clinical practice. Taken together, the application of evidence‐based treatments on a consistent guidelines‐driven basis combined with a uniform definition of treatment response characteristics may enable safe de‐escalation of therapy while minimizing the risk of relapse. Ultimately, the utilization of biomarkers may be most helpful for understanding which therapeutic may be most likely to succeed or induce a durable remission in an individual patient.

## 6 CIDP, a Call for Clearer Definitions

The absence of clear and consistent definitions for key terms concerning the treatment response, such as “disease activity”, “response,” “refractory,” “relapse,” and “remission” is a recurring unmet need in the CIDP landscape. This shortcoming leads to inconsistencies in treatment decisions between clinicians, confusion among patients, and difficulty interpreting results from clinical trials.

To address these ambiguities, the GBS/CIDP Foundation International has assembled a task force of patients and clinicians that aims to develop consensus‐driven definitions of these key terms (personal communication). Upon completion, it is anticipated that the standardized definitions will be available for use in clinical care and clinical trials. This harmonized terminology may not only facilitate more accurate clinical assessments but also promote more robust and comparable research outcomes, ultimately improving the care of individuals with CIDP.

## 7 Conclusion

This report highlights some of the critical unmet needs in the management of CIDP. By identifying gaps in current diagnostic and therapeutic practices as well as measuring the treatment response, we hope to promote innovative initiatives that aim to address these needs.

Presently, the most pressing unmet needs include: (1) standardized definitions key for the management of CIDP, (2) an enhanced understanding of minimal changes in outcome measures that can be applied to individual patients, (3) treatment management protocols that give clearer guidance on immunotherapy optimization, and (4) biomarkers that can facilitate the diagnostic process and objectively identify disease activity. A CIDP definitions Task Force of patients and expert clinicians, aimed at mitigating some of these unmet needs, has been assembled, and we anticipate proposed definitions for key terms in the near future. The existing CIDP guidelines remain the best resource by which treatment decisions and treatment escalation can be followed. Innovative studies that can provide data supporting the best approach to optimization and appropriateness of immunotherapies when first‐line options are not successful are encouraged. Biomarker research remains an active area of ongoing research. While some candidate biomarkers (e.g., neurofilament light chain [32]) hold promise, a reliable biomarker that captures disease activity in the majority of patients remains elusive.

By understanding the diverse experiences and challenges faced by healthcare providers worldwide, we hope to work toward more effective strategies that address the specific needs of CIDP patients globally.

## Author Contributions

**Jeffrey A. Allen:** supervision, validation, visualization, writing – review and editing. **Helmar C. Lehmann:** supervision, validation, visualization, writing – review and editing. **Eduardo Nobile‐Orazio:** supervision, validation, visualization, writing – review and editing. **Luis Querol:** supervision, validation, visualization, writing – review and editing. **Yusuf A. Rajabally:** supervision, validation, visualization, writing – review and editing.

## Conflicts of Interest

J.A.A.: Annexon, Alexion, CSL Behring, Takeda, Grifols, Argenx, Sanofi, Immunovant, Immunoabs, Alnylam, AstraZeneca, Dianthus. H.C.L.: LFB, Grifols, CSL Behring, Argenx. E.N.‐O.: Personal fees for Advisory or Scientific Boards from ArgenX, Belgium; Takeda, Italy; Takeda, USA; CSL‐Behring, Italy; CSL‐Behring, USA; Janssen, USA; Kedrion, Italy; LFB, France; Roche, Switzerland; Sanofi, USA. L.Q.: Instituto de Salud Carlos III—Ministry of Economy and Innovation, Spain, CIBERER, GBS‐CIDP Fundation International, ArgenX, UCB, Sanofi, Annexon, Alnylam, Lycia Therapeutics, CSL Behring, Johnson and Johnson, Merck, Novartis, Roche, Takeda, Sanofi‐Genzyme, Dianthus, Avilar, Nuvig, Peripheral Nerve Society. Y.R.: LFB, ArgenX, Sanofi, Takeda, Dianthus, Janssen, Grifols, CSL Behring.

## Data Availability

The data that support the findings of this study are available on request from the corresponding author. The data are not publicly available due to privacy or ethical restrictions.
